# Image processing techniques to estimate weight and morphological parameters for selected wheat refractions

**DOI:** 10.1038/s41598-021-00081-4

**Published:** 2021-10-25

**Authors:** Rohit Sharma, Mahesh Kumar, M. S. Alam

**Affiliations:** Department of Processing and Food Engineering, Punjab Agricultural University, Ludhiana, Punjab India

**Keywords:** Optical imaging, Scientific data, Statistics

## Abstract

The geometric and color features of agricultural material along with related physical properties are critical to characterize and express its physical quality. The experiments were conducted to classify the physical characteristics like size, shape, color and texture and then workout the relationship between manual observations and using image processing techniques for weight and volume of the four wheat refractions i.e. sound, damaged, shriveled and broken grains of wheat variety PBW 725. A flatbed scanner was used to acquire the images and digital image processing method was used to process the images and output of image analysis was compared with the actual measurements data using digital vernier caliper. A linear relationship was observed between the axial dimensions of refractions between manual measurement and image processing method with R^2^ in the range of 0.798–0.947. The individual kernel weight and thousand grain weight of the refractions were observed to be in the range of 0.021–0.045 and 12.56–46.32 g respectively. Another linear relationship was found between individual kernel weight and projected area estimated using image processing methodology with R^2^ in the range of 0.841–0.920. The sphericity of the refractions varied in the range of 0.52–0.71. Analyses of the captured images suggest ellipsoid shape with convex geometry while the same observation was recorded by physical measurements also. A linear relationship was observed between the volume of refractions derived from measured dimensions and calculated from image with R^2^ in the range of 0.845–0.945. Various color and grey level co-variance matrix texture features were extracted from acquired images using the open-source Python programming language and OpenCV library which can exploit different machine and deep learning algorithms to properly classify these refractions.

## Introduction

Image processing techniques are being used in the area of post harvest handling of agricultural produce for ensuring quality and hygiene of raw and processed food fit for human consumption. The four selected physical characteristics are vital parameter in assessing the quality of food. The consumer demands the agricultural produce to be of uniform size, characteristic shape and identical size. A clear, fully mature and undamaged kernel appearance is regarded as first index of good quality produce.

Computer vision technology has immense potential to use characteristics like size, shape, color and texture attributes from digital images to characterize the agricultural produce. Wheat (*Triticum aestivum* L.) is an important cereal crop. The storage and milling operations have a distinct effect on quality of grains. The proper inspection and classification of good quality grains is of great importance. The varieties, quality and purity inspection of grains are more difficult and complicated. In present grain handling and milling system, types of grain and quality is mostly visually assessed. This evaluation process is tedious and not fully reliable. The grain assessment with manual methods are challenging even for trained people. The main difficulty arise in the training of these evaluators. Also, the time required for quality evaluation of these grains is comparatively higher, which thwarting quick decision making and large-scale evaluation. Grain quality pricing depends on quality indices based on end-use requirements. The physical characteristics such as size, shape, color and texture play an important role in expressing grain quality along with classifying kernels into damaged, discoloured and infested kernels^1^. The use of computer vision is already common in many areas such as image recognition patterns, product quality analysis, fault prediction models and fraud detection^2^.

Many researchers have developed methods to elucidate varietal dissimilarities in grains using machine vision systems based on seed size and colour characteristics of the sample. These techniques have been used to identify and classify food grains based on kernel variety, type, refractions and insect infestation. A classification methodology was given to segregate kernels like wheat, oats and rye based on color and texture parameters^3^.

A rapid assessment of head rice yield based on computer vision systems (CVS) as a tool was conducted^4,5^. The relationships describing the whiteness of milled rice was presented that can improve rice quality. In another study, the bran layer area on surface of rice was determined the using digital image analysis^6^. Some studies have also showed various techniques to classify products as different grades based on kernel size and shape variations^7^. The rice kernels were classified into short, long, slender, round and bold grades using a support vector machine classifier using variations in length, width and its ratio of individual seeds as the classification criteria. Based on relative differences in kernel sizes, the proposed technique could also differentiate head rice from brokens and brewers^8^. In another study, the visual grading of soybean was described using image analysis techniques taking size uniformity as the classification criteria^9^. Few studies have been carried out to identify whole and broken fractions in wheat, rice, corn and soybeans^10–13^. Additionally, a method was developed to detect the presence of fissures in rice kernels using machine vision and image processing that would result in lesser head rice yields during milling^14^. Few researchers studied the effect of changing imaging backgrounds (mostly white and black) for better object identification^15,16^. A classification criteria for barley, wheat and rice was developed based on grain shape variations for better pattern recognition using image processing^17,18^. Some studies were conducted based on variations in kernel colour to differentiate between different pulses^19,20^. The accuracies of varietal classification have been reported as high as 99%; with process time in few seconds^21^. However, it is well known seed colour changes in certain lentils due to oxidation reactions. Therefore colour as the sole feature would not provide accurate classification for pattern recognition. Many studies have been reported for varietal classifications of grains with high accuracies by extracting morphological^22–24^, colour^25,26^ and textural features^27^ and their combinations^28,29^. The combination of colour, morphological and textural features were used for classification of dockage and foreign matter^30^. A classification methodology was reported for insect damaged wheat kernels identification in bulk^31,32^ along with identification of adult insect pests using captured images^33^. Some studies suggested use of variations in colour space to identify discoloured and chalky kernels by machine vision systems^34^. A classification model was developed to identify fungal damages in soybean kernels based on multivariate discriminant analysis was developed using Red, Green, Blue (RGB) color space with acceptable levels of accuracy^35^. A handheld device was developed for classification of Indian basmati rice into healthy and discoloured kernels^36^. Further, a methodology was proposed for identifying maturity by studying variations in colour of paddy using RGB colour space features as the classification criteria^37^. The current study is based on extracting quantifiable information from the digital images for different quality refractions of wheat grains^38^ with the use of digital images.

The weight, geometric and color parameters of kernels are an important parameter for assessment of quality of grains. The present study focuses on to estimate weight, geometric and color parameters of different refractions of wheat through image process techniques and to develop relationship between the images and the physical parameters of these refractions.

## Materials and methods

**Figure 1:**
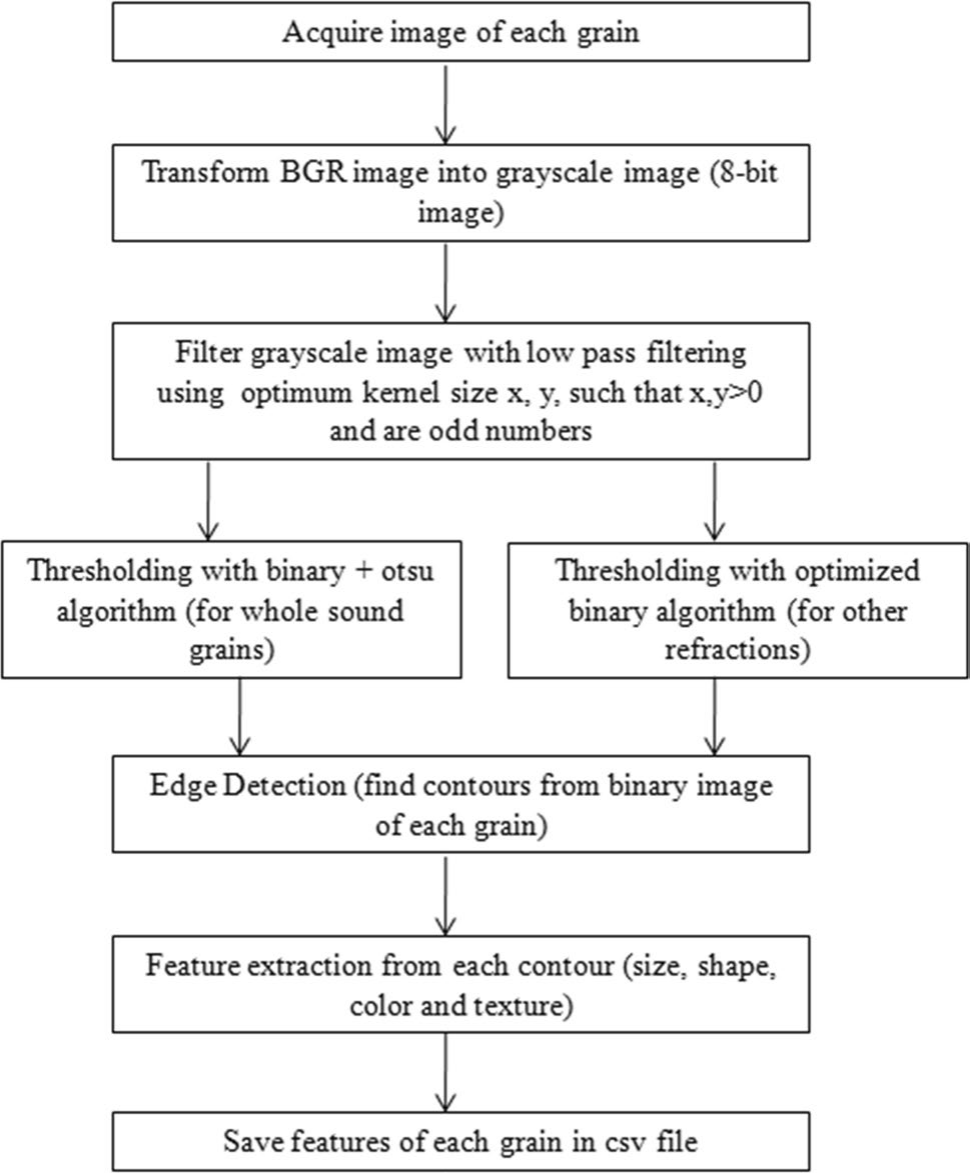
Flow diagram for features extraction from acquired images.

The samples of wheat variety PBW 725 were collected from Punjab Agricultural University, Ludhiana experimental fields. The procedures adopted in study are in compliance with Indian Standard IS 4333 Part 1(1996) guidelines. The kernels were grouped into four categories such as sound grains, damaged grains, shriveled grains and broken grains. The randomly selected one hundred kernels from each category were manually placed on the scanner. The images are acquired using a flatbed scanner model Cannon scan 5600F with CCD 6-line color with white fluorescent light source. All the captured images were of 24-bit color format at 200 dots per inch (dpi) resolution. Black background was used for all the images as it gives more contrast to wheat color and nullify the shadow effect. The images were saved as non compressible bitmap images (bmp format). The computer algorithm was developed using open-source Python language and OpenCV library to extract size, shape, color and textural parameters for each kernel (Fig. 1). First, the color picture (BGR format) was converted into three 8-bit grayscale images i.e. blue, green and red bands. After applying the low pass Gaussian filter of 5 × 5 kernel size for removing small discrepancies in the image, the combined thresholding algorithm of binary and Otsu operator was applied to perform the background segmentation using opencv threshold function with combined flag of binary and OTSU thresholding techniques^39^. It is an automatic thresholding technique developed to segment the desired object from background image, i.e., whole sound wheat grain in this case. For other refractions i.e. damaged, shriveled and broken grains, the binary thresholding algorithm was used to background segmentation^40^. The threshold value is selected based on the results of the histogram analysis and, was invariable for the same environment conditions. The threshold values of pixels was calculated using equation given below for damaged, shriveled and broken grains refractions (dominated the area of gray levels) less than 37, 74 and 35 respectively for proper segmentation and took the shape of normal distribution. The next process involves using a mask and bitwise operation over all the other pixels that do not lie in our described range of pixels.

1 $$dst\left(x,y\right)= \left\{\begin{array}{ll}maxval&\quad if\, src\left(x,y\right)>thresh\\ 0&\quad otherwise\end{array}\right.$$

where src(x,y) and dst(x,y) refers to the intensity of pixels(x,y) of source and destination images respectively. Later, edge detection of each kernel was accomplished by detecting contours for each binary image of grain using OpenCV CHAIN_APPROX_NONE function for non compression of pixels and stores all boundary points. The size, shape, color and texture features were extracted from each contour(cnt) and the whole data was saved in csv/html file. For the other dimensional measurement of extracted image, the minimum bounding rectangle (MBR) of each contour was determined. The MBR (ferret diameter) is the minimum rectangle with the smallest area which encloses desired contour. Two dimensional measurement of the kernels are taken as width and height of the MBR in this study.

The computer algorithm was programmed in Python programming language version 3.7 using the OpenCV 4, Numpy, Scikit-Image and Scipy scientific computing libraries^41^. OpenCV image processing library^42^ was used for reading and pre-processing of images, color space conversions and image features extraction based on size, shape and color. Scipy image processing library^43^ was used in extracting statistical features like mean, standard deviation, skewness and kurtosis from the images. Scikit-Image image processing library^44^ was used for the extraction of Gray-level co-occurrence matrix features (GLCM texture features) from the images. All algorithm development steps and experiments were conducted on a personal computer (Intel Core 2 Duo i7 2.70 GHz with 6 Gbytes of RAM).

The physical properties like axial dimensions, equivalent diameter, sphericity, roundness and weight of each kernel were estimated. Weight of each kernel was measured using a sensitive electronic weighing scale (Citizen CY220, USA) with an accuracy of 0.001 g. A digital vernier caliper (make Mitutoyo) having 0.01 mm least count was used to determine the axial dimensions of each kernel.

The arithmetic mean diameter (D_a_) and geometric mean diameter (D_g_) of the kernels has been calculated by considering spherical shape for a grain^45^.

2 $$\mathrm{Da}=(\mathrm{L}+\mathrm{W}+\mathrm{T})/3$$

3 $$D_{g} \,\, = \,\,\,\left( {L \times W \times T} \right)^{1/3}$$

where: L, W, T are length, width and thickness of the sample (mm) respectively.

The sphericity (Sp) can be described as the ratio geometric mean diameter to major dimension of sphere kernels; while roundness indicates the sharpness of the corners to detect spherical, oblate, regular and oblong shapes was determined using following formulas^45^

4 $$Sphericity\,\,\, = \,\,\,\frac{{Geometric\,\,mean\,\,dia(D_{g} )}}{Major\dim ension}$$

5 $$Roundness\begin{array}{*{20}c} {} \\ {} \\ \end{array} ratio\,\, = \,\,\,\frac{L}{{\sqrt {W \times T} }}$$

Thousand grains weight was computed by weighing 100 kernels using electronic weighing scale (Citizen CY220, USA) with least count 0.001 and then estimated mass of 1000 kernels by factor of 10. This is usually denoted by gm per 1000 kernels^46,47^.

The surface area is one of the significant physical properties of the kernels and can be related to respiration rate, colour evaluation and heat transfer studies in heating and cooling processes. It was calculated by the following relation^48^.

6 $$\mathrm{Sa }= \frac{{\mathrm{\pi BL}}^{3}}{2\mathrm{L}-\mathrm{B}}$$

7 $$B={(\mathrm{W}*\mathrm{T})}^{1/2}$$

where, Sa is surface area in mm^2^ and B is lateral geometric mean diameter.

The principle dimensions of length and width were used to calculate the volume of the different kernels considering them prolate and oblate spheroid. The following formulas were use in its calculations

8 $${\text{Volume of prolate spheroid }} = \frac{4}{3}\pi \times L \times W^{2} \; {\text{mm}}^{{3}}$$

9 $${\text{Volume of oblate spheroid }} = \frac{4}{3}\pi \times L^{2} \times W\; {\text{mm}}^{{3}}$$

The shape of the kernels was also estimated using aspect and ellipsoid ratios. Aspect ratio (A.R.) and Ellipsoid ratio (E.R.) has been defined^49,50^ as:

10 $${\text{A}}.{\text{R}}. \, = {\text{ a }}/{\text{ b}}, \, \left( {{\text{A}}.{\text{R}}. \, \ge { 1}.0} \right)$$

11 $${\text{E}}.{\text{R}}. \, = {\text{ b }}/{\text{ c}}, \, \left( {{\text{E}}.{\text{R}}. \, \ge { 1}.0} \right)$$

where a, b, c are major, intermediate and minor diameters, respectively (mm).

### Feature extraction using image processing

**Figure 2:**
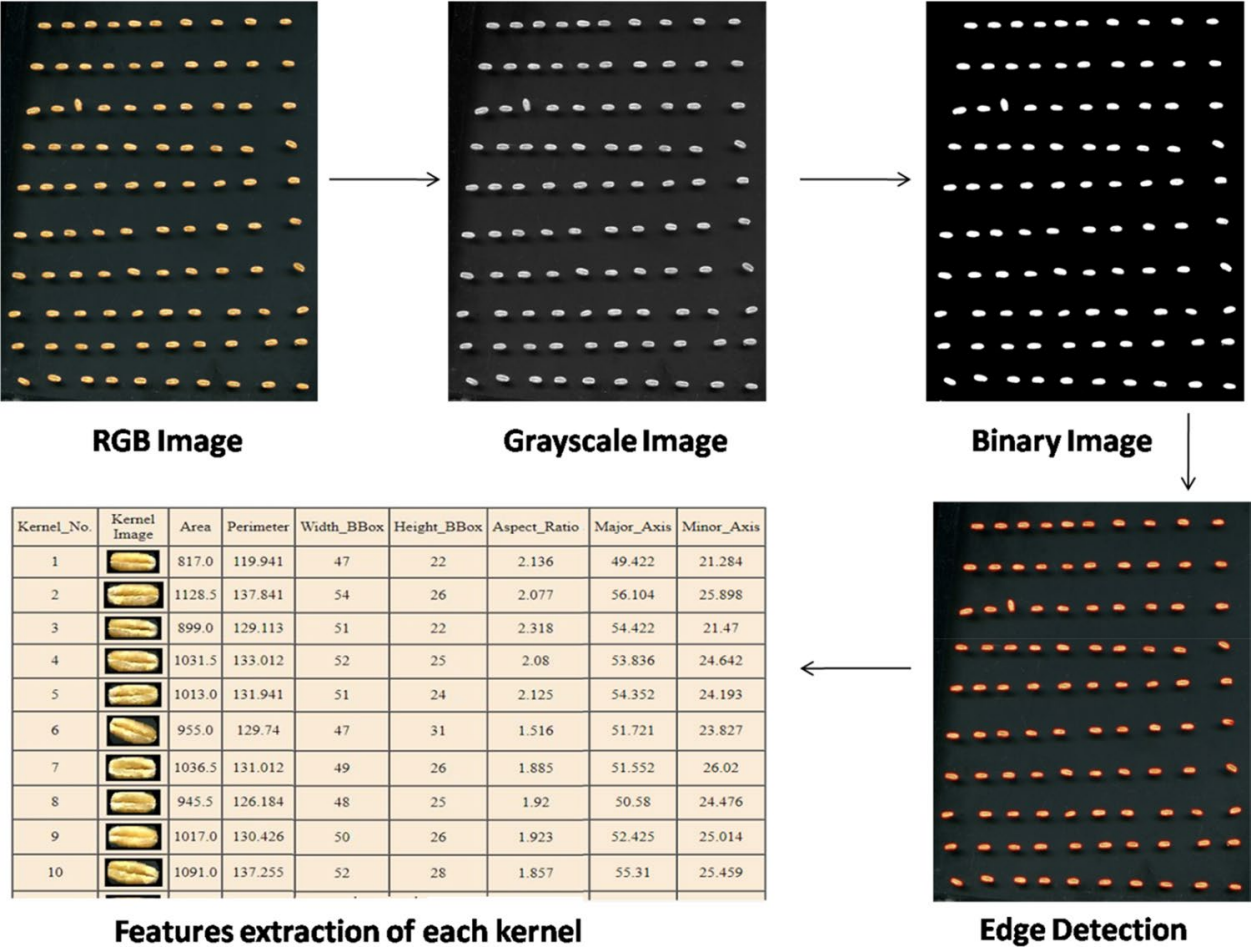
Flow diagram for features extraction from acquired images.

**Figure 3:**
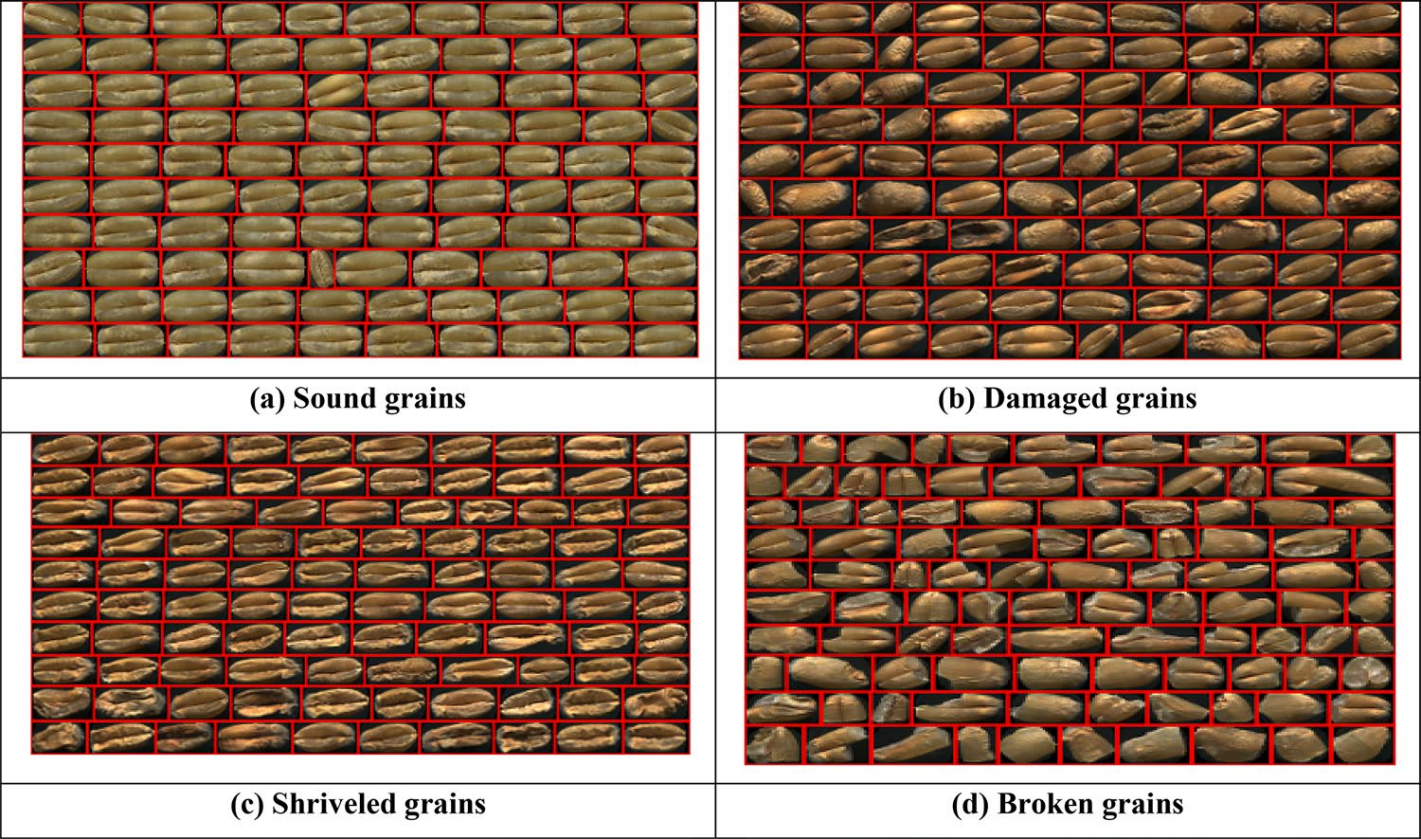
Segmented images of wheat refractions **(a)** sound grains, **(b)** damaged grains, **(c)** shriveled grains, **(d)** broken grains.

A total of 30 features related to size and shape (15 features), color (9 features) and texture (6 features) of the selected kernels has been estimated using the python software. A list of extracted features using OpenCV library has been given in Table 1. All the functions in the library are loaded into the algorithm using 'cv2' command. A flow diagram for features extraction from acquired images is given in Fig. 2. One hundred segmented images of each refractions extracted using image processing software are shown in Fig. 3.

**Table 1:** List of size and shape features extracted from images using OpenCV library function.

Extracted feature	Function used^40,42^
Minimum bounding rectangle	cv2.boundingRect(cnt)
Area (A)	cv2.contourArea
Perimeter (P)	cv2.arcLength(cnt, True)
Solidity	cv2.contourArea/float(hull_area)
Eccentricity, minor diameter (m), major diameter (M)	cv2.fitEllipse(cnt)

### Size and shape estimation

One hundred kernels from each refraction i.e. sound grains, damaged grains, shriveled grains and broken grains having different sizes were chosen from a given lot based on its visual appearance. The shape of the selected kernels and its size has been computed using the open-source Python software. The parameters relating the size and shape of the object were broadly selected to describe the geometric make up of the kernels. The features like minimum bounding rectangle (MBR), area (A), perimeter (P), solidity, minor diameter (m), major diameter(M) were estimated from the image analysis. These features were extracted using different functions available in OpenCV library (Table 1). The derived features from the image analysis is given in Table 2. The brief explanation of these parameters is as follows:

**Table 2:** Size and shape derived features from image analysis.

Derived feature	Equation used^47,54^
Boundary rectangle fill (extent)	$\frac{{\text{Pixel count}}}{{\text{(height *width)}}}$
Bounding rectangle to perimeter	$\frac{{{\text{Perimeter}}}}{{{\text{2*(height }} + {\text{ width)}}}}$
Equivalent diameter (Feret diameter)	$2\sqrt{\frac{A}{\pi }}$
Circulation factor	P/$\pi$
${\text{Compactness}}$	$\frac{4\pi A}{{P}^{2}}$
Elongation	$\frac{\mathrm{M}-\mathrm{m}}{\mathrm{M}+\mathrm{m}}$
Aspect ratio	L/B
Ratio of surface area to cubic volume	A/M^3^

#### Area

It is the calibrated area of the image. It is measured as number of pixels in the image and it is converted into the mm^2^ by multiplying with a calibration factor. The calibration factor was calculated on the basis of scanning resolution of 200 dpi.

#### Major and minor axis

Major and minor axis of minimum bounding rectangle (MBR) in pixels which is converted into mm using calibration factor.

#### Volume

Volume of the kernels is worked out using major and minor axis derived from formula for prolate and oblate spheroid objects.

#### Bounding rectangle fill

It signifies how much an image shape matches to a rectangular pattern. It is expressed as the ratio of pixel count of filled MBR to the calibrated area of image worked out by multiplying the height and width of the image. Its value is one for a completely rectangular shape.

#### Bounding rectangle to perimeter

It is the ratio of perimeter of region of interest of image to the perimeter of bounding rectangle. For convex shape, the interior angle between the outline is always less than 180 degree and its value is one.

Solidity is the ratio of contour area to its convex hull area. The convex hull area is the smallest possible set of pixels enclosing the shape. The solidity value varies from 0 to 1.

Equivalent diameter or Feret diameter of a circular object has the same area as the computed kernels.

Circulation factor is expressed as diameter of the circle with a perimeter equal to the perimeter of the kernel.

#### Compactness

It is the ratio of area of object to the area of square. It describes the resemblance of the object to square shape. When the seed is a circle, compactness is equal to 1, for a square seed, it is (π/4 = 0.78) and as the value approaches zero, it indicates an increasingly elongated polygon.

Elongation refers to the difference between the lengths of the major and minor axes of the best fit ellipse divided by the sum of lengths. It is zero for circle and one for long and narrow ellipse.

#### Aspect ratio

It is the ratio of the height to its width inside MBR. The log_10_ of this ratio gives symmetric measure of aspect ratio.

### Color and texture estimation

The color analysis was carried out for each kernel. The 24-bit captured images are processed in open-source Python image processing software. All the images were imported into python Integrated Development Environment (IDE) as a three dimensional array with size [x, y, 3] (where x denotes rows, y denotes columns and 3 denote the red, green and blue channels). For each category of refractions, red (R), green (G), and blue (B) color densities were extracted by OpenCV library using python language. All the three channels consists of two-dimensional array with the size of 'x' rows and 'y' columns. Since the imported files are of 24-bit color images, all the three separated channels would be 8-bit grayscale images with the integer values ranging between 0 and 255. H, S and V channels were also separated by converting the original RGB image to HSV image using BGR2HSV function in OpenCV library. The standard Royal Horticultural Society Color Chart (RHSCC) was selected to describe nearest color of scanned kernels^51–53^. An algorithm was developed to find the mean distance of color (sRGB values) between the RHSCC and scanned images by euclidean distance formula after converting sRGB values into Numpy arrays in python. The extracted color features are given in Table 3. Among the different bases possible for the color representation, the Hue-Saturation-Value (HSV) model has potential to our problem since this model is closer to human color perception than RGB.

**Table 3:** Color features extracted from image analysis.

Feature extracted	Function used^42,44,54–56^
Mean red, mean green and mean blue channels	np.array(cv2.mean())
Normalized red–green differential index ($${NDI}_{rg})$$	$$\frac{\left|R-G\right|}{R+G}$$
Normalized red–blue differential index ($${NDI}_{rb})$$	$$\frac{\left|R-B\right|}{R+B}$$
Normalized green–blue differential index ($${NDI}_{gb})$$	$$\frac{\left|G-B\right|}{G+B}$$
Hue, saturation and value (HSV)	cv2.cvtColor(bgr image, cv2.COLOR_BGR2HSV)
GLCM texture features	greycomatrix() and greycoprops()

The cv2.mean function returns the mean red(R), green(G), blue(B) channel in BGR format. All three mean colors are retrieved by converting function values into using numpy array function(np.array). HSV values were obtained from BGR image by using cv2.cvtColor() function. The six GLCM texture features were obtained using functions greycomatrix() and greycoprops() from skimage library.

The color based texture features like gray-level co-occurrence matrix (GLCM) were extracted using greycomatrix function from skimage library. The GLCM texture features include contrast, dissimilarity, homogeneity, angular second moment(ASM), energy and correlation (Table 4). The statistical analyses is carried out by using descriptive statistic parameters available in Microsoft Excel 2007.

**Table 4:** GLCM Texture features extracted from image analysis.

Feature extracted	Function used
Contrast	$\sum\nolimits_{i,j = 0}^{levels - 1} {P_{i,j} } (i - j)^{2}$
Dissimilarity	$\sum\nolimits_{i,j = 0}^{levels - 1} {P_{i,j} } \left| {i - j} \right|$
Homogeneity	$\sum\nolimits_{i,j = 0}^{levels - 1} {\frac{{P_{i,j} }}{{1 + (i - j)^{2} }}}$
Angular second moment (ASM)	
Energy	$\sum\nolimits_{i,j = 0}^{levels - 1} {P^{2}_{i,j} \sqrt {ASM} }$
Correlation	$\sum\nolimits_{i,j = 0}^{levels - 1} {P_{i,j} \left[ {\frac{{(i - \mu_{i} )(j - \mu_{j} )}}{{(\sigma_{i}^{2} )(\sigma_{j}^{2} )}}} \right]}$

Where P[i,j,d,theta] is an 4-D ndarray grey level co-occurence matrix and represents a histogram of co-occuring greyscale values at a given offset over an image. The P value represents a matrix with value number of times that grey-level j occurs at a distance d and at an angle theta from grey-level i.

The syntax for function greycomatrix, P[i,j,d,theta] is given below. The default values were used for these parameters.

P[i,j,d,theta] = skimage.feature.texture.greycomatrix(image, distances, angles, levels = 256, symmetric = false, normed = false).

## Results and discussion

### Physical characteristics of the selected refractions

A perusal of the Table 5 indicates the important axial dimensions of the selected refractions of wheat. The three average axial dimensions vary between 4.91 and 6.20 mm lengthwise, 2.47–3.43 mm width wise and 2.24–2.87 mm thickness wise. All the refractions are geometrically convex with both aspect ratio and ellipsoid ratio more than one and can be best described as ellipsoid shaped rather than sphere. The values of ratios L_avg_/T_avg_ and W_avg_/T_avg_ more than one indicate scalene type of ellipsoid.

**Table 5:** Axial dimensions of different grain fractions of selected wheat variety (n = 100).

Characteristics	Sound grain	Damaged grain	Shriveled grain	Broken grain
Avg. length (L_avg_), mm	6.2 ± 0.65	5.82 ± 2.04	5.62 ± 1.15	4.91 ± 2.45
Avg. width (W_avg_), mm	3.43 ± 0.43	3.04 ± 1.58	2.47 ± 1.07	3.16 ± 1.21
Avg. thickness (T_avg_), mm	2.87 ± 0.41	2.57 ± 1.5	2.24 ± 0.8	2.59 ± 0.94
Geometrical mean (D_g_), mm	3.94 ± 0.43	3.56 ± 1.57	3.24 ± 0.95	3.4 ± 1.03
Arithmetic mean (D_a_), mm	4.17 ± 0.42	3.81 ± 1.54	3.64 ± 0.88	3.55 ± 1.05
Lateral geometric mean (D_l_), mm	3.14 ± 0.4	2.79 ± 1.51	2.35 ± 0.86	2.86 ± 1.03
L_avg_/W_avg_ (aspect ratio)	1.81 ± 0.28	1.97 ± 1.47	2.56 ± 0.74	1.58 ± 1.41
L_avg_/T_avg_ (ellipsoid ratio)	2.16 ± 0.31	2.36 ± 2.12	2.79 ± 0.84	1.94 ± 1.96
L_avg_/D_g_	1.58 ± 0.17	1.66 ± 0.83	1.92 ± 0.34	1.44 ± 0.82
W_avg_/T_avg_ (ellipsoid ratio)	1.2 ± 0.15	1.2 ± 0.65	1.1 ± 0.48	1.23 ± 0.17

The other physical characteristics describing weight and shape features of the refractions have been presented in the Table 6. The thousand grain weight of the selected refractions varies between 12.56 and 46.32 g. The roundness ratio and sphericity values indicate the ellipsoid shape of all the refractions. The average single kernel weight of the refractions varied from 0.021 to 0.045 g. The information regarding the surface area and volume of prolate and oblate spheroid shape is very important in differentiating and classification of different types of kernel refractions.

**Table 6:** Physical characteristics of different grain fractions of selected wheat variety (n = 100).

Characteristics	Sound grain	Damaged grain	Shriveled grain	Broken grain
Sphericity	0.64 ± 0.08	0.61 ± 0.21	0.52 ± 0.11	0.71 ± 0.27
Roundness ratio	1.98 ± 0.3	2.15 ± 1.77	2.67 ± 0.69	1.75 ± 1.66
Individual kernel weight (g)	0.045 ± 0.01	0.036 ± 0.03	0.022 ± 0.03	0.021 ± 0.03
Surface area of prolate spheroid (Sp), mm^2^	232.54 ± 47.7	194.06 ± 119.23	161.6 ± 96.65	175.3 ± 104.98
Surface area of oblate spheroid (So), mm^2^	348.83 ± 69.23	302.99 ± 177.18	311.36 ± 122.03	240.58 ± 178.82
Volume of prolate spheroid (Vp), mm^3^	307.67 ± 91.19	236.86 ± 192.02	163.46 ± 180.38	210.19 ± 201
Volume of oblate spheroid (Vo), mm^3^	555.28 ± 157.59	445.27 ± 327.91	407.56 ± 316.64	334.7 ± 390.7
Volume of ellipsoid (V_e_), mm^3^	256.64 ± 75.89	200.58 ± 167.36	146.83 ± 161.51	171.85 ± 172.82
Thousand grain weight (g)	46.32	35.64	23.9	12.56

### Characteristics of size and shape of wheat refractions by image processing

**Figure 4:**
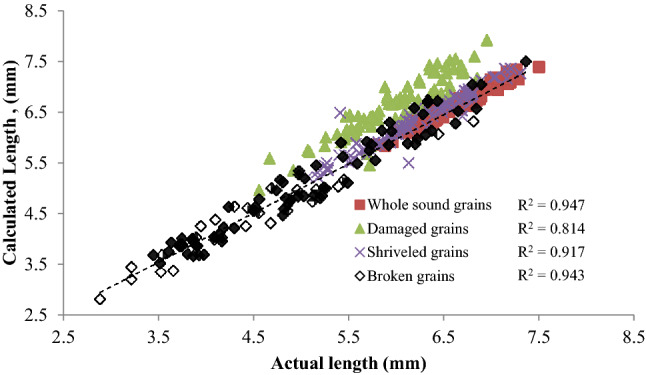
Relationship between the length of refractions measured with caliper and the calculated from image.

**Figure 5:**
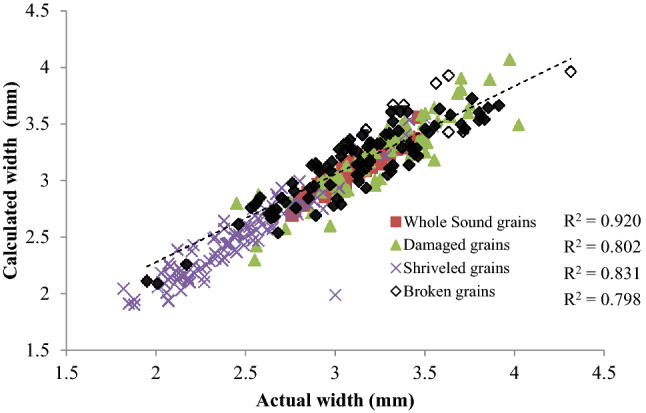
Relationship between the width of refractions measured with caliper and the calculated from image.

The relationship between the length and width of selected refractions and the number of pixels as obtained from the image analysis is linear with R^2^ in the range of 0.81–0.95 and 0.80–0.92 respectively (Figs. 4, 5). Hence the images of refractions acquired with flatbed scanner at 200 dpi resolution can be used. The important parameters describing the shape and size of the refractions through its image are presented in Table 7. The Bounding rectangle fill and bounding rectangle to perimeter indicate that the calibrated area of the image may be considered as out bulging type shape without any indent i.e., convex geometry with resemblance to rectangle by 73–78%. The compactness values for the refractions lie in the range of 0.67–0.77 so the calibrated areas cannot be assumed as square. The elongation parameter of refractions varies between 0.2–0.44 indicating that the shape of sound grains, damaged and shriveled grains is better considered as elliptical in comparison to brokens. The ratio of the area evaluated by counting the pixels in the image to area of MBR fill containing the image of refractions lie in the range of 0.74–0.77 indicating the effectiveness in its acquisition and processing (Table 7). Hence along with size, image processing can be used to provide useful information about the shape of the refractions.

**Table 7:** Size and shape parameters derived from image to express the shape of selected wheat refractions.

Characteristics	Sound grain	Damaged grain	Shriveled grain	Broken grain
Area (px^2^)	989.3 ± 223.8	1001.29 ± 369.21	756.86 ± 453.15	729.78 ± 498.22
Perimeter (px^2^)	129.43 ± 14.66	131.4 ± 38.68	118.36 ± 26.21	108.59 ± 38.55
Solidity	0.98 ± 0.02	0.97 ± 0.08	0.97 ± 0.04	0.96 ± 0.06
Major diameter (px^2^)	52.24 ± 6.27	51.53 ± 13.29	49.88 ± 8.86	38.63 ± 20.39
Minor diameter (px^2^)	24.41 ± 3.59	25.1 ± 7.01	19.58 ± 8.25	25.04 ± 8.58
Boundary rectangle fill	0.78 ± 0.12	0.74 ± 0.12	0.74 ± 0.11	0.73 ± 0.15
Bounding rectangle to perimeter	0.86 ± 0.04	0.86 ± 0.24	0.87 ± 0.04	0.85 ± 0.07
Feret diameter	35.46 ± 4.24	35.58 ± 7.01	30.88 ± 8.37	30.23 ± 9.59
Circulation factor	41.2 ± 4.67	41.83 ± 12.31	37.68 ± 8.34	34.57 ± 12.27
Compactness	0.74 ± 0.05	0.73 ± 0.32	0.67 ± 0.11	0.77 ± 0.23
Elongation	0.36 ± 0.07	0.34 ± 0.14	0.44 ± 0.13	0.2 ± 0.28
Aspect ratio	1.94 ± 1.29	1.8 ± 0.91	2.18 ± 0.71	1.33 ± 1.29
Ratio of surface area to cubic volume	0.007 ± 0	0.007 ± 0	0.006 ± 0	0.014 ± 0.02
MBR fill	0.77 ± 0.12	0.74 ± 0.12	0.74 ± 0.11	0.74 ± 0.15

### Weight estimation of selected wheat refractions

**Figure 6:**
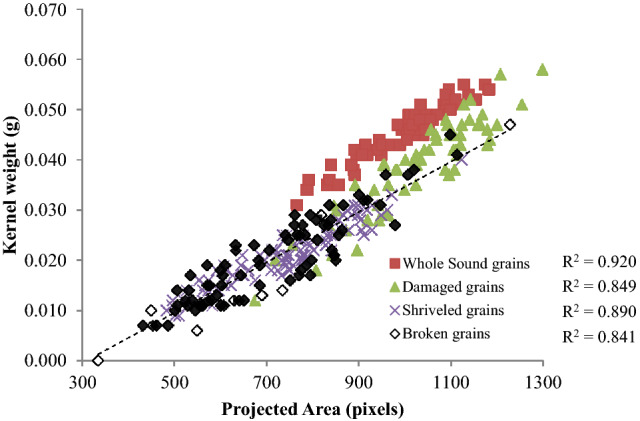
Relationship between the individual kernel weight of refractions measured with weighing balance and the calculated from image.

**Figure 7:**
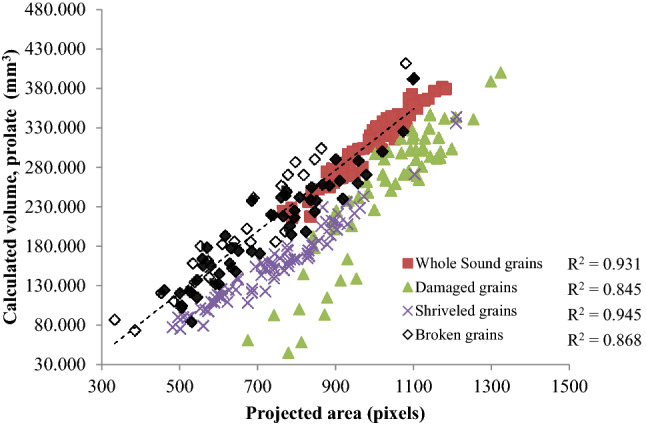
Relationship between the volume of refractions derived from measured dimensions and the calculated from image.

Weight of the kernels is an important parameter currently being used in physical quality evaluation of kernels. It indicates soundness and wholesomeness of kernel. Using machine vision technology, a relationship was established between the weight and volume of the kernel with the calibrated area estimated from the acquired images using machine vision technology (Fig. 6). A plot between the weight and calibrated area suggest linear relationship with R^2^ value varies from 0.84 to 0.92. Sound grains indicate good correlation followed by Shriveled grains. Considering prolate spheroid shapes of the kernels, a plot between volume estimated using Mohesnin’s equivalent diameter and calibrated area suggested a linear relationship with R^2^ value varies from 0.84 to 0.94 (Fig. 7). Here, shriveled and sound grains showed good correlation. Thus machine vision system has good potential for application in online quality estimation during wheat handling, packaging and storage operation.

### Characteristics of color and texture features of wheat refractions by image processing

The red, green and blue channels were separated from 24-bit captured images of all wheat refractions. The mean and normalized differential indexes for red, green and blue channels were calculated (Table 8). The shriveled grains are having maximum values for all three channels while broken grains are having least values for these channels. As such, the variation in color is subjected to number of factors and exact match is not possible when images are acquired with two different systems. So, an algorithm was developed to find the closest matching color of acquired images with standard charts. The closest color code between the standard RHSCC and scanned images was calculated as 148A, 165A, 199B and N199A for sound grains, damaged grains, shriveled grains and broken grains respectively (Table 8). The values for NDI_rg,_ and NDI_gb_ were calculated for all the refractions. The maximum values of NDI_rg,_, NDI_rb_ and NDI_gb_ correspond to shriveled grains, brokens grains and sound grains whereas minimum values of NDI_rg,_ correspond to sound grains, and minimum value for NDI_rb_ and NDI_gb_ was correspond to shriveled grains (Table 8). The HSV values for all refractions are also estimated from the algorithm using OpenCV library^42^ and the values are shown in Table 8.This information can be very helpful in classification of these different refractions. The color based texture features (GLCM) were extracted from images using skimage library. The values for texture features such as contrast, dissimilarity, homogeneity, angular second moment(ASM), energy and correlation are given in Table 8. The contrast, dissimilarity and energy texture value for broken grains is maximum at 2269.7, 31.61 and 0.04 whereas these values are least for sound grains. The homogeneity texture value is highest for sound grains and minimum for shriveled grains. The correlation texture value also varies from 0.49 to 0.65 and is maximum for shriveled grains and minimum for broken grains. These color and texture features clearly showed the variation in values for all these refractions and certainly will be helpful in classification and identification of these kernels using different machine learning and deep learning algorithms.

**Table 8:** Color and texture parameters derived from image of selected wheat refractions.

Characteristics	Sound grain	Damaged grain	Shriveled grain	Broken grain
RHSCC closest color code	148A	165A	199B	N199A
Mean red	119.79 ± 18.79	114.59 ± 44.59	141.15 ± 22.85	101.96 ± 22.96
Mean green	104.98 ± 16.98	91.82 ± 37.82	117.19 ± 17.81	83.27 ± 19.73
Mean blue	69.62 ± 12.62	66.53 ± 43.47	90.26 ± 13.74	56.96 ± 21.04
NDI_rg_	0.07 ± 0.01	0.11 ± 0.05	0.09 ± 0.02	0.1 ± 0.03
NDI_rb_	0.27 ± 0.04	0.27 ± 0.14	0.22 ± 0.05	0.28 ± 0.08
NDI_gb_	0.2 ± 0.03	0.17 ± 0.09	0.13 ± 0.04	0.19 ± 0.05
Value (lightness)	116.15 ± 17.14	111.72 ± 41.03	139.47 ± 18.52	96.2 ± 18.77
Saturation	103.97 ± 11.98	103.03 ± 40.47	91.11 ± 17.04	99.75 ± 16.64
Hue	29.23 ± 6.6	30.73 ± 12.71	31.9 ± 11.9	33.34 ± 10.47
Contrast	1166.83 ± 608.2	1550.96 ± 1602.6	1606.83 ± 1071.4	2269.7 ± 1912.5
Dissimilarity	21.05 ± 8.59	25.91 ± 12.42	25.72 ± 8.65	31.61 ± 13.92
Homogeneity	0.09 ± 0.04	0.07 ± 0.04	0.07 ± 0.05	0.08 ± 0.05
Angular second moment	0.001	0.001	0.001	0.001
Energy	0.03 ± 0	0.03 ± 0.01	0.03 ± 0.01	0.04 ± 0.05
Correlation	0.56 ± 0.14	0.62 ± 0.28	0.65 ± 0.17	0.49 ± 0.43

## Conclusion

Image processing technique was used to estimate weight, geometric and color parameters of four different types of the wheat refractions with the help of a flatbed scanner. The open-source Python image processing software was used to obtain their size, shape, volume, color and texture features. A linear relationship was observed between the axial dimensions of refractions between manual measurement and image processing method with R^2^ in the range of 0.798–0.947. The individual kernel weight and thousand grain weight of the refractions were observed to be in the range of 0.021–0.045 and 12.56–46.32 g respectively. Another linear relationship was found between individual kernel weight and projected area estimated using image processing methodology with R^2^ in the range of 0.841–0.920. The sphericity of the refractions varied in the range of 0.52–0.71. A linear relationship was observed between the volume of refractions derived from measured dimensions and calculated from image with R^2^ in the range of 0.845–0.945. Further, nine color and six GLCM texture features were also estimated which can exploit different machine and deep learning algorithm to properly classify these refractions for accurate identification of kernel conditions.
